# Web-Based 24-Hour Dietary Recall Tool for Russian Adults and School-Aged Children: Validation Study

**DOI:** 10.2196/41774

**Published:** 2023-08-16

**Authors:** Sandrine Pigat, Mariya Soshina, Yulia Berezhnaya, Ekaterina Kryzhanovskaya

**Affiliations:** 1 Creme Global Dublin Ireland; 2 PepsiCo, Inc Moscow Russian Federation

**Keywords:** dietary assessment, 24-hour dietary recall, extent of agreement, energy and nutrient intake, Russian diet, interviewer-administered, web-based self-administered, diet, food intake, dietary recall, energy intake, nutrient intake

## Abstract

**Background:**

Data on dietary intakes in Russian adults and children are assessed very infrequently primarily due to the time, cost, and burden to the participants for assessing dietary patterns. To overcome some of those challenges, the use of web-based 24-hour recall methods can be successfully used.

**Objective:**

The study objective is to assess the extent of agreement between a self-administered and an interviewer-administered 24-hour dietary recall in Russian adults and school-aged children using an adaptation of a web-based 24-hour recall tool.

**Methods:**

This web-based dietary assessment tool is based on a previously validated tool, which has been adapted to the Russian diet and language. A randomized 50% (n=97) of 194 participants initially completed a self-administered web-based dietary recall, followed by an interviewer-administered 24-hour dietary recall later that same day, and vice versa for the other 50% (n=97) of participants. Following at least 1 week wash-out period, during visit 2, participant groups completed the 2 dietary recalls in the opposite order. Statistical analysis was carried out on the intake results from both methods for the 2 recalls. Finally, an evaluation questionnaire on ease-of-use of the tool was also completed.

**Results:**

In total, intakes of 28 nutrients and energy were analyzed in this study. The Bland-Altman analysis showed that between 98.4% and 90.5% of data points were within the limits of agreement among all age groups and nutrients analyzed. A “moderate to excellent” reliability between the 2 methods was observed in younger children. In older children, a “moderate to good” reliability was observed, with the exception of sodium. In adults, “moderate to excellent” reliability between both methods was observed with the exception of vitamins B1, B2, and B6, and pantothenic acid. The level of agreement between the categorization of estimates into thirds of the intake distribution for the average of the 2 days was satisfactory, since the percentages of participants categorized into the same tertile of intake were ˃50%, and the percentages of participants categorized into the opposite tertile of intake were <10%. The majority of respondents were very positive in their evaluation of the web-based dietary assessment tool.

**Conclusions:**

Overall, the web-based dietary assessment tool performs well when compared with a face-to-face, interviewer-administered 24-hour dietary recall and provides comparable estimates of energy and nutrient intakes in Russian adults and children.

**Trial Registration:**

ClinicalTrials.gov NCT04372160; https://clinicaltrials.gov/ct2/show/NCT04372160

## Introduction

In this data-driven era, we are moving toward the digitalization of data gathering, which has proven to be very effective in dietary intake assessments [1-5]. Web-based tools create the opportunity to make data gathering more scalable and frequent, without the burden on the researcher and the participants of paper-based approaches.

Methods to collect dietary data via web-based tools are increasingly used in health surveys, due to their convenience, efficacy, and flexibility. Web-based self-administered 24-hour dietary recalls can provide the opportunity for more efficient and cost-effective dietary assessments in comparison to traditional paper-based methods [6-9]. Techniques such as the use of food portion size photographs, the multiple-pass method [10], linked food databases, and smart food data searches make it less onerous to carry out small- and large-scale intake studies. A web-based 24-hour dietary recall questionnaire tool was used in this study to assess intakes in the Russian population, including children. It has been demonstrated to accurately record food intake in the Irish population [11,12]. Food diaries are generally more burdensome to the participant, and hence, they can have a lower completion rate. This study used the 24-hour recall method as it has moderate burden on the participant, and it is a widely used method in dietary intake studies [13]. In this study, we are comparing an adapted version of the aforementioned web-based self-administered 24-hour dietary recall with a trained interviewer-administered 24-hour dietary recall in Russian children and adults as a validation of the web-based tool.

The current state of dietary consumption and nutrition status in the Russian population is difficult to measure due to the large, diverse geography of Russia and associated food cultures of different regions. The Russia Longitudinal Monitoring Survey-Higher School of Economics (RLMS-HSE) [14] has been conducted by the National Research University Higher School of Economics and ООO Demoscope with Carolina Population Center, University of North Carolina at Chapel Hill, and the Federal Center of Theoretical and Applied Sociology of the Russian Academy of Sciences via several phases over the past 30 years. These data have been collected using paper-based collection methods during face-to-face interviews on demographics, food intake, and health status. Due to the amount of work involved in data collection, RLMS-HSE 24-hour dietary intake data are not published very often and only cover a narrow range of nutrients. More recently, in 2013 and 2018, Rosstat (Federal State Statistics Service) conducted large-scale nutrition surveys in all constituent entities of the Russian Federation, which were also carried out according to a unified methodology for collecting, processing, and reporting actual data developed at the Federal Research Center of Nutrition, Biotechnology and Food Safety, the same used in RLMS-HSE study [15].

This is the most current data available on dietary intake in Russia. Therefore, more regular and extensive analysis of food and nutrient intakes is needed in the Russian population, including adults and children. With a very large and diverse population, digital methods for capturing dietary intakes, which are easy to use, validated, and with low participant burden, will be beneficial. The adaptation of this tool consisted in using local databases on traditional foods and food portion sizes consumed in Russia. In addition, the tool was extended to assess children’s dietary intakes (via a proxy) and adapted to the Russian language. The primary objective of this study was to assess the extent of agreement of nutrient intakes between the web-based self-administered and the interviewer-administered 24-hour dietary recalls.

## Methods

### Tool Adaptation

The previously validated web-based dietary assessment tool [11,12] has been adapted to capture the Russian diet by incorporating photographs to ascertain small, medium, and large (and in between) portion sizes in adults and children of commonly consumed foods, including composite dishes and beverages by Creme Global and PepsiCo Holdings, LLC. The tool was also adapted to the Russian language with the assistance of native-speaker translators. The web-based intake assessment tool consists of independent components that facilitate the collection of dietary intake data without direct interaction with a researcher. These components include a demographic questionnaire, 2 nonconsecutive 24-hour dietary recalls following the multiple pass method [10], a food frequency questionnaire, alongside supplementary questionnaires on anthropometry and health status, and finally a tool evaluation questionnaire. All these stages occur at predetermined time points and have been developed independently of each other, meaning different parts of the tool could potentially be used depending on the requirements of any given survey or study. A food database of typical Russian foods was created by IPSOS (marketing insights agency) and PepsiCo Holdings, LLC. IPSOS created a list of typical Russian foods and portion sizes depicted by photographs (purchased and weighed) for use in the web-based dietary assessment tool. The final database resulted in 890 unique food or beverage items and 124 food groups and 721 photos with standard portion sizes. PepsiCo collected nutritional data (energy; macronutrients: protein, carbohydrate, total fat and fatty acids, and dietary fiber; and micronutrients: vitamins A, D, E, C, and B and calcium, magnesium, iron, phosphorus, copper, zinc, potassium, and sodium) using the *Chemical Composition and Caloric Content of Russian Food* reference book [16] and data from other available sources [17].

### Recruitment

Study participants (males and females between 7 and 61 years of age) were recruited via 3 commercial medical centers in Saint Petersburg by contacting and inviting patients from their databases over the period from February 19, 2020, to October 12, 2020. Participants signed an informed consent form and were not included if they had any disease or condition that required chronic therapeutic nutritional treatment, were on a diet, had any formal training in nutrition, or had prior experience completing dietary recalls. They were also excluded if they were unable to complete computer-based dietary questionnaires due to mental, physical, or visual limitations. A sample size of 204 participants (68 participants per age subgroup: younger school-age children [7-13 years old], adolescents [14-17 years old], and adults [18-65 years old]) was deemed acceptable since it was comparable with the number of participants recruited in several similar investigations [9,18-20]. Similar sampling was done in previous pilot studies [11]. For the younger school-age children, parents were used as a proxy to complete recalls and questionnaires. Participants who withdrew from this study were replaced via additional recruitment to maintain the required numbers for the study. In total, 194 participants completed this study.

### Ethics Approval

We hereby declare all ethical standards have been respected during this study. Ethical approval for this study was obtained through the Independent Interdisciplinary Committee on the Ethical Review of ClinicalTrials.gov (and was also registered on ClinicalTrials.gov NCT04372160).

All participants read a participant information leaflet and signed an informed consent form. For children 7-13 years old, a parent or a caregiver signed the informed consent form. If the participant was an adolescent 14-17 years old, both the adolescent and the parent or caregiver signed the informed consent form. A participant information leaflet consisted of the study approval, the purpose of this study, inclusion and noninclusion criteria, a detailed description of the research procedure, the questionnaires and data analyses included in this study, participant’s rights and responsibilities, confidentiality of participant’s personal information, and sponsor’s and investigator’s contacts. Participants had enough time to familiarize themselves with the documents and could ask any questions and discuss it with the investigator before giving their agreements.

For the participants’ identity protection, the study investigator assigned every participant a unique code, such as a series of numbers or letters. All data from the participant were collected using a corresponding assigned code, not his or her name. All data that were available for sponsor and contract research organization were blinded. Every participant was given compensation in the form of reimbursement for transportation expenses to and from the investigational site.

### Data Collection

Dietary recall data using the web-based 24-hour dietary recall and an interviewer-administered 24-hour dietary recall were collected on the same day for 2 nonconsecutive days 1 week apart. Both methods (web-based self-administered and interviewer-administered food recall) used the same food database and food pictures to capture portion sizes.

The interviewer-administered 24-hour dietary recall was based on the methodology used by Rosstat (composed by Martinchik et al [21]). The interviewers were trained to gather precise, detailed, and accurate information to reduce error and bias. The reference method was used according to the guidelines of the Federal Research Center for Nutrition, Biotechnology and Food Safety. They were familiar with the dietary patterns of the respondents, had a list of foods commonly eaten by the target population and were familiar with composite dishes, their recipes, and preparation methods, and were aware of how food is served. They were trained on how to use standard probes and prompts properly, how to measure portion size, particularly for mixed dishes, and how to ask questions in a nonjudgmental and noninfluential manner during the recall. Each participant was interviewed separately. For school-age children 7-13 years old, parents acted as proxy and provided answers for their children during the interview. For the interviewer-administered 24-hour dietary recall, participants recalled information about all food and drinks consumed the previous day (from midnight to midnight). A printed version of the portion sizes incorporated into the 24-hour dietary recall component of the web-based dietary assessment tool was used to aid portion size quantification.

The data were entered directly by the investigator into a web system for clinical trials provided by DataMATRIX (Data MATRIX EDC/IWRS). The intake of each nutrient was calculated for each product or dish based on the portion size (in grams) assessed and recorded by the interviewer on the electronic case report form and food list containing nutrient composition per 100 g and summed up by recall. Web-based self-reported data including the 24-hour recall were entered directly by the participant into the web-based assessment tool developed by Creme Global, Dublin, Ireland. The macronutrients and micronutrients for each food for the web-based tool were automatically calculated and provided directly in the export.

Participants were randomized (n=97, 50%) to either complete the self-administered web-based dietary assessment tool or interviewer-administered 24-hour dietary recall first, with the order reversed on the second observation day. At the end of this study, each participant additionally completed an evaluation questionnaire about the ease of use of the web-based tool. After participants completed all assessments in visit 2, participants were given a study incentive.

### Statistical Analysis

Analyses were performed using SAS statistical software (version 9.4; SAS Institute) for Microsoft Windows operating system statistical software. As a primary analysis, Bland-Altman analysis was used to assess the extent of agreement between self-reported and interviewer-administered outputs for nutrient intakes. The 2 methods of dietary assessment were considered comparable if >95% of the data were within the limits of agreement. However, in light of recent developments in the best practices for conducting and interpreting studies to validate self-reported dietary assessment methods [22], several tests in combination were evaluated as a secondary analysis. To assess relationships between nutrient intake estimates for each recall day of the web-based self-administered and interviewer-administered recalls, Spearman correlation coefficient was calculated. As a measure of reliability, reflecting both degree of correlation and agreement between measurements, the nutrients were evaluated on a continuous scale rather than categorically. Weighted κ is usually used to assess agreement [22,23]. The κ coefficient does not take into account the degree of disagreement between methods, and all disagreement is treated equally as total disagreement. It also does not indicate whether the agreement or lack thereof is because of a systematic difference between the 2 methods or because of random differences (error because of chance).

Nutrient intake distributions were tested for normality for both web-based self-administered and interviewer-administered recalls on both days using the Shapiro-Wilk test. The differences in intake between the 2 methods were tested. Depending on whether the intake distribution was normally distributed, an appropriate comparison test (paired *t* test or Wilcoxon signed rank test) was applied to analyze the significant differences between estimates of nutrient intakes between web-based self-administered and interviewer-administered recalls 1 and 2.

## Results

### Demographics

Overall, 204 participants (68 participants per age subgroup) were recruited for this pilot study, 194 participants completed this study and had available data on the 2 assessments by the 2 methods. Table 1 summarizes the demographic characteristics of those participants. The mean age in younger school-age children was 9.7 (SD 1.9) years, in older school-age children was 15.6 (SD 1.0) years, and in adults was 34.4 (SD 10.1) years.

**Table 1:** Demographic characteristics.

	Younger school-age children (7-13 years; N=63)	Older school-age children (14-17 years; N=64)	Adults (18-65 years; N=67)
Male, n (%)	33 (52)	33 (52)	33 (49)
Female, n (%)	30 (48)	31 (48)	34 (51)
Age (years), mean (SD)	9.7 (1.9)	15.6 (1.0)	34.4 (10.1)

### Levels of Agreement Between Web-Based and Interviewer-Administered Recalls—Primary Analysis

**Figure 1:**
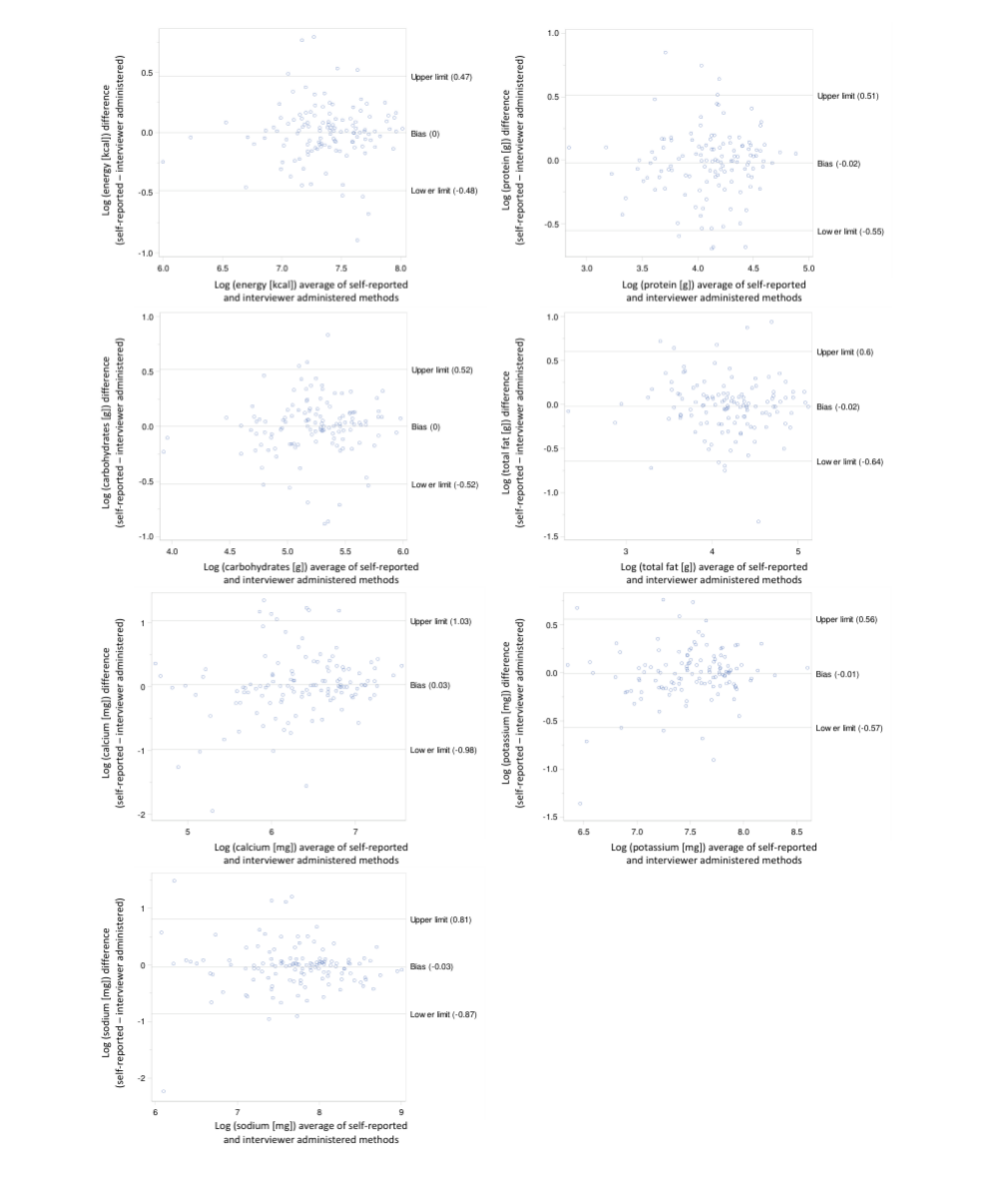
Bland-Altman plots for nutrient intakes of younger school-age children (N=63) for the 2 methods including both days of measurements.

**Figure 2:**
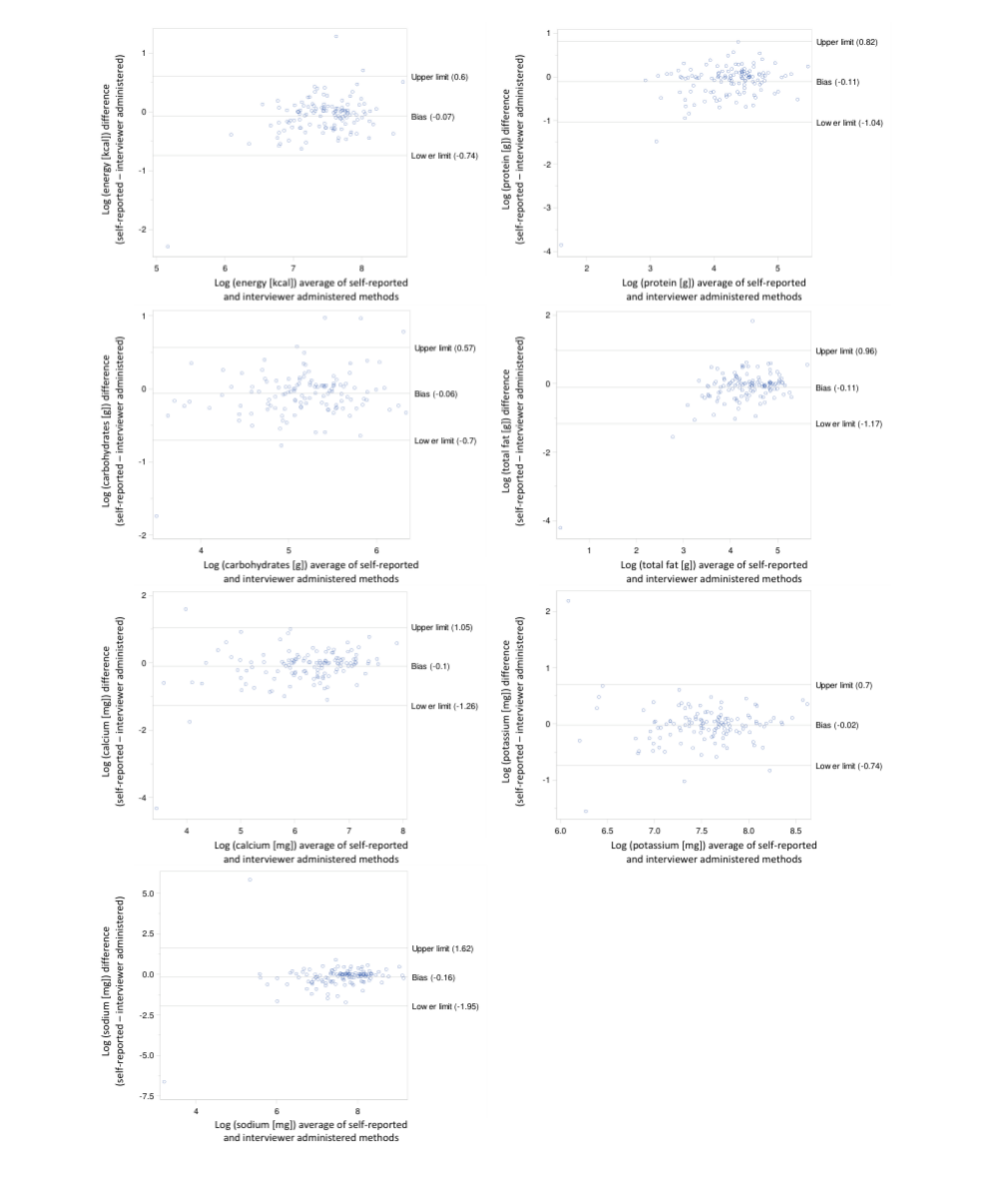
Bland-Altman plots for nutrient intakes of older school-age children (N=64) for the 2 methods including both days of measurements.

**Figure 3:**
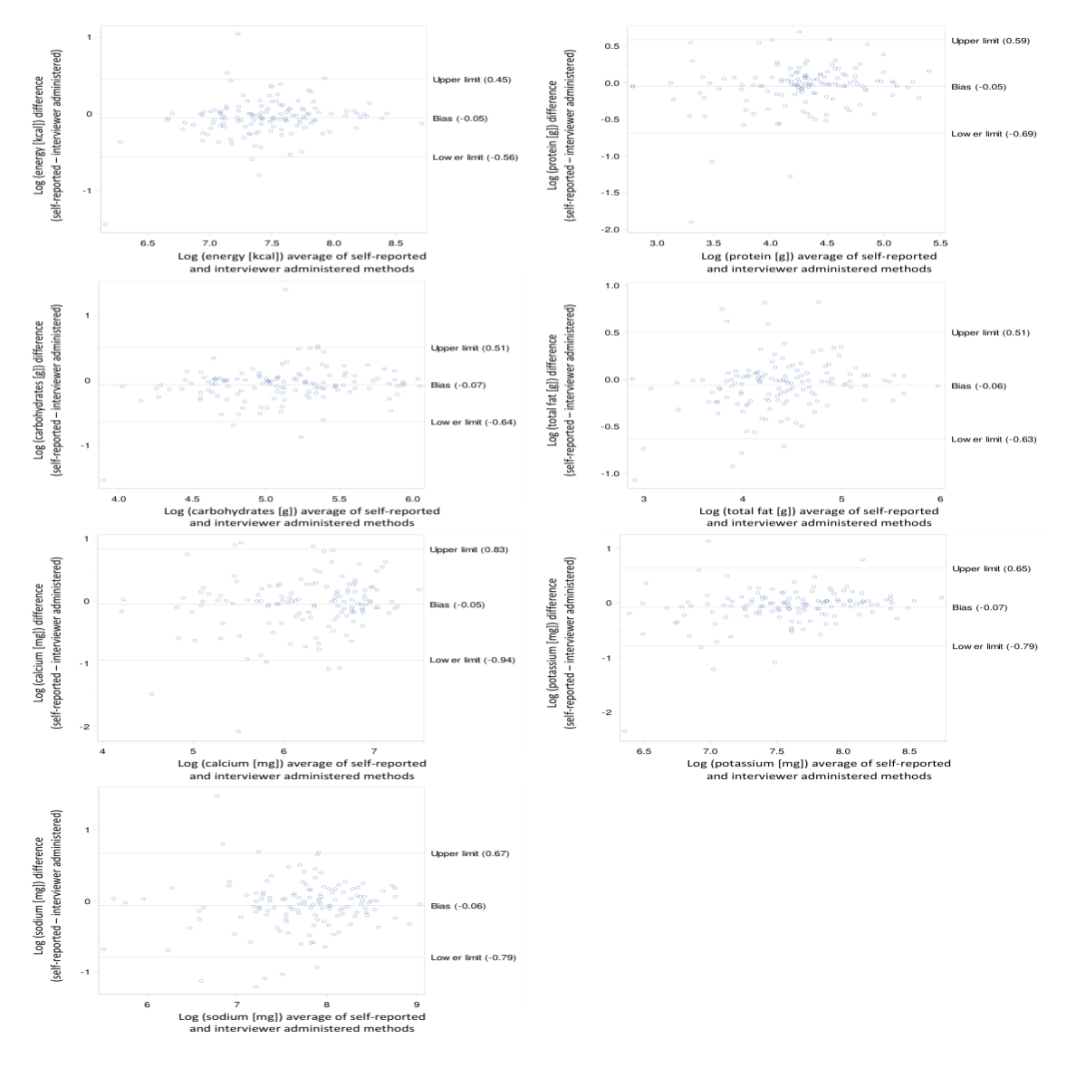
Bland-Altman plots for nutrient intakes of adults (N=67) for the 2 methods including both days of measurements.

Most of the Bland-Altman plots for the differences in energy, protein, carbohydrate, fat, potassium, calcium, and sodium and nutrient intakes were within the limits of agreement (Figures 1-3). In the younger school-age children, the percentage of the nutrients within the limits of agreement ranged from 9.5% (vitamin E) to 3.2% (vitamin B1; Table 2). The percentage of data points outside the limits of agreement for the older school-age children and adults, respectively, ranged from 8.6% (retinol) to 1.6% (sodium) and from 8.2% (vitamin D, folate, and zinc) to 3.0% (vitamin C, B2, niacin, and pantothenic acid). Overall, the Bland-Altman analysis showed agreement in all age groups. The Bland-Altman plots only show a few gross outliers, which may indicate unfamiliarity with the technique, differences in nutrient coding, or possible missing dishes. No significant bias was identified for any nutrient.

**Table 2:** Bland-Altman analysis of agreement for energy and nutrient between interview administered and web-based recall in all 3 cohorts.

Nutrient	Percentage within limits of agreement		
	Younger school-age children	Older school-age children	Adults
Energy (kcal)	92.9	97.7	95.5
Protein (g)	93.7	98.4	95.5
Total sugar (g)	92.9	94.5	94.0
Carbohydrates (g)	92.1	95.3	96.3
Dietary fibers (g)	92.9	94.5	97.0
Total fat (g)	92.1	97.7	92.5
Saturated fat (g)	94.4	95.3	96.3
Polyunsaturated fat (g)	95.2	95.3	94.0
Retinol vitamin A (mcg)	94.4	91.4	94.8
β-Carotene (mcg)	93.7	95.3	92.5
Vitamin D (mcg)	91.3	93	91.8
Vitamin E (mg)	90.5	95.3	92.5
Vitamin C (mg)	95.2	93	97.0
Vitamin B1 (mg)	96.8	95.3	95.5
Vitamin B2 (mg)	93.7	96.1	97.0
Niacin (mg)	96	95.3	97.0
Pantothenic acid (mg)	93.7	93	97.0
Vitamin B6 (mg)	94.4	93	94.8
Biotin (mcg)	92.9	96.1	92.5
Folate (mcg)	92.1	93	91.8
Vitamin B12 (mcg)	92.1	92.2	94.0
Calcium (mg)	90.5	97.7	94.0
Magnesium (mg)	94.4	95.3	96.3
Iron (mg)	92.9	94.5	95.5
Phosphorus (mg)	92.1	96.9	96.3
Copper (mcg)	95.2	93	94.8
Zinc (mcg)	95.2	93.8	91.8
Potassium (mg)	92.9	96.9	95.5
Sodium (mg)	94.4	98.4	93.3

### Levels of Agreement Between Web-Based and Interviewer-Administered Recalls—Secondary Analysis

The results for the association between the estimates of nutrient intake using the web-based self-administered 24-hour dietary recall and the interviewer-administered 24-hour dietary recall split by recall number and percentage observation in the same tertile can be found in Tables S1 to S3 in Multimedia Appendix 1. In younger school-aged children, for both recall 1 and 2, a moderate (ρ 0.5-0.7) to very high (ρ 0.9-1.0) correlation between the estimates of daily nutrient intake from each method was observed [24]. In older school-age children, a moderate (ρ 0.5-0.7) to high (ρ 0.7-0.9) correlation between the estimates of daily nutrient intake from each method was observed, and in adults, a moderate (ρ 0.5-0.7) to very high (ρ 0.9-1.0) correlation between the estimates of daily nutrient intake from each method was observed (Multimedia Appendix 1). For both recall 1 and 2, a moderate (values between 0.5 and 0.8) to excellent (values >0.9) [25] reliability between both methods was observed in young school-age children. In older school-age children, a moderate (values between 0.5 and 0.8) to good (values between 0.8 and 0.9) reliability between both methods was observed. The only exception was poor reliability for sodium (mg)—the intraclass correlation coefficient (ICC) value for that nutrient during recall 2 was 0.5. In adults, a moderate (values between 0.5 and 0.8) to excellent (values >0.9) reliability between both methods was observed. Poor reliability was observed for vitamin B1 (mg) and vitamin B2—the ICC values for those nutrients during recall 1 were both 0.4, respectively. During recall 2, poor reliability was observed for vitamin B1 (mg), pantothenic acid (mg), and vitamin B6 (mg)—the ICC values for those nutrients were 0.3, 0.5, and 0.4, respectively.

According to the results of cross-classification analysis, conducted for the average of the 2 days, the level of agreement between the categorization of estimates into thirds of the nutrient intake distribution was good, since the percentages of participants categorized into the same tertile of intake were ˃50%.

### Energy and Nutrient Intakes

Tables S4 to S6 in Multimedia Appendix 1 present the estimated daily energy and nutrient intakes for both recall days and using both intake methods across the 3 age groups. In younger school-age children, nutrient intakes between the 2 methods for recall 1 and 2 were not statistically different. In older school-aged children, significantly lower intakes were found when using the web-based self-administered method for protein, carbohydrates, niacin, and sodium. In adults, lower intakes were found when using the web-based self-administered method for energy, total sugar, carbohydrate, dietary fiber, total fat, vitamin C, vitamin B1, magnesium, iron, phosphorus, copper, and zinc.

### Food Diary Evaluation

The results of the user acceptability assessment of the web-based dietary assessment tool based on the evaluation questionnaire data are summarized in Table 3. The majority of participants found the navigation of the tool, determining the portion size, and adding or removing foods from their diet were either easy or neither complicated nor easy. One of the questions was about forgotten food and drinks. The most frequent answer mentioned drinks across all age groups.

**Table 3:** Food Diary evaluation broken down by age group and number of participants.

		Younger school-age children (N=63)	Older school-age children (N=64)	Adults (N=67)
**In general, I consider using the Food Diary app is...**		n=62	n=63	n=65
	Very complicated	1 (2)	0	1 (2)
	Complicated	0	2 (3)	9 (14)
	Neither complicated nor easy	39 (63)	32 (51)	37 (57)
	Easy	16 (26)	23 (37)	12 (19)
	Very easy	6 (10)	6 (10)	6 (9)
**I find that determining the serving size of my dish with the serving size in the photo was...**		n=0	n=63	n=65
	Very complicated	N/A^a^	0	1 (1.5)
	Complicated	N/A	4 (6)	10 (15)
	Neither complicated nor easy	N/A	34 (54)	33 (51)
	Easy	N/A	20 (32)	16 (25)
	Very easy	N/A	5 (8)	5 (8)
**Adding or removing food from my daily diet was...**		n=0	n=63	n=65
	Very complicated	N/A	1 (2)	1 (2)
	Complicated	N/A	1 (2)	7 (11)
	Neither complicated nor easy	N/A	35 (56)	39 (60)
	Easy	N/A	23 (37)	17 (26)
	Very easy	N/A	3 (5)	1 (2)
**What foods or drinks did you forget to record?^b^**		n=62	n=63	n=65
	Drinks	9 (14)	13 (20)	13 (19)
	Main dishes	2 (3)	0	0
	Snacks	4 (6)	9 (14)	6 (9)
	Eating out	5 (8)	2 (3)	1 (2)

^a^N/A: not applicable.

^b^Multiple answers are possible.

## Discussion

### Principal Findings

The central objective of this study was to assess the extent of agreement between a web-based self-administered and an interviewer-administered 24-hour dietary recall, assessed on the same day for 2 nonconsecutive days 1 week apart. This is in accordance with guidance on the EU Menu methodology [26], which states that the day of the week for data collection should, as far as possible, be randomly assigned, and the time between the 2 nonconsecutive data collection days should be at least 7 days apart. Originally, it was suggested that 2 methods of dietary assessment should be considered comparable if more than 95% of the data plots would lie within the limits of agreement. In light of recent developments for conducting and interpreting studies to validate self-reported dietary assessment methods [22], several tests were interpreted in combination to provide insight into the properties of the method being evaluated.

Despite the wide limits of agreement for most micronutrients including vitamins (especially polyunsaturated fat, retinol vitamin A, β-carotene, vitamin C, vitamin D, vitamin B1, vitamin B12, copper, and zinc), Bland-Altman plots indicate only a few gross outliers, which may indicate differences in nutrient coding, portion sizes, and missing dishes. It is argued that although the Bland-Altman analysis is relatively simple to execute, it does not distinguish adequately between fixed and proportional bias [27]. A major advantage of the web-based dietary assessment tool and of similar web-based dietary assessment tools is the reduced cost associated with the collection of dietary intake data compared with traditional methods. More importantly, web-based methodologies facilitate the collection of data in a neutral environment, in the absence of a researcher with less burden for the participant, which may encourage participants to report intake more honestly.

Due to the findings of this study, the web-based dietary assessment tool could be considered for future studies to collect dietary intake data in the Russian population. Furthermore, the methods developed within this study were applied and validated in both adults and children, enabling the examination of intakes at a family level using the same methodology. Younger school-aged children had a proxy to fill out their questionnaire, which is consistent with the methodology used in the current National Health and Nutrition Examination Survey data collection (1999 to present). Participant acceptability data gathered suggest the web-based dietary assessment tool was well received by most participants in this study sample, which indicates the potential of the web-based dietary assessment tool for use in nutrition-related research in the Russian Federation.

### Strengths and Limitations

The majority of participants recruited as part of this study were young (about 3 quarters of the participants in the adult cohort were younger than 38 years), healthy, and motivated individuals, and therefore may not represent the general adult population with respect to their ability to use the self-administered web-based dietary assessment tool and their preference of dietary assessment methods. However, similar studies examined recruitment in very specific settings, such as university participants [11]. Overall, this work is unique; it is the first time that a web-based dietary 24-hour recall tool has been used and validated for assessing the diet in Russian adults and children. The tool has been adapted from a previous study in the Russian language and eating habits, which includes traditional foods, photographs with portion sizes, and respective food composition data of energy, and 28 nutrients. Moreover, the tool is capturing the diet of younger and older children, and the results were comparable to the interviewer-administered recall. This pilot study shows that this web-based 24-hour recall tool can be used for future Russian population studies.

### Conclusions

The results obtained in this study demonstrate that the web-based dietary assessment tool performed well when compared to a face-to-face interviewer-administered 24-hour dietary recall, with comparable estimates of energy and nutrient intakes. Overall, the usability of the tool was easy for most participants in this study.

## Appendix

Multimedia Appendix 1 Association between the estimates of nutrient intake using the online self-administered 24-hour dietary recall and the interviewer-administered 24-hour dietary recall.

## Abbreviations

ICC: intraclass correlation coefficient
RLMS-HSE: The Russia Longitudinal Monitoring Survey-Higher School of Economics

## Data Availability

The data sets generated and analyzed during this study are property of PepsiCo Inc and are not publicly available but are available from the corresponding author upon reasonable request.
