# Supplementary material for: Web-Based 24-Hour Dietary Recall Tool for Russian Adults and School-Aged Children: Validation Study
Source: JMIR Form Res. 2023 Aug 16;7:e41774. doi: 10.2196/41774 (PMC10468702; doi:10.2196/41774)
Supplement: Multimedia Appendix 1 [file formative_v7i1e41774_app1.docx]

Table S1. Association between the estimates of nutrient intake using the online self-administered 24-hour dietary recall and the interviewer-administered 24-hour dietary recall split by recall number and % observation in the same tertile (average of the two days). Cohort: Younger school-age children (N=63).

|  | Recall 1 | | | Recall 2 | | | Observations in the same tertile |
| --- | --- | --- | --- | --- | --- | --- | --- |
| Nutrient | Geometric mean ratio [95% CI] | Spearman correlation coefficient (rho) | Intra-class correlation coefficient | Geometric mean ratio [95% CI] | Spearman correlation coefficient (rho) | Intra-class correlation coefficient | % |
| Energy (Kcal) | 0.98 [0.93, 1.04] | 0.798 | 0.847 | 1.02 [0.95, 1.09] | 0.659 | 0.691 | 71 .4 |
| Protein (g) | 0.98 [0.92, 1.04] | 0.749 | 0.771 | 0.99 [0.92, 1.06] | 0.661 | 0.74 | 61.9 |
| Total sugar (g) | 0.94 [0.85, 1.03] | 0.747 | 0.762 | 1.00 [0.90, 1.10] | 0.787 | 0.732 | 69.8 |
| Carbohydrates (g) | 0.99 [0.93, 1.05] | 0.747 | 0.792 | 1.02 [0.95, 1.09] | 0.693 | 0.737 | 63.5 |
| Dietary fibers (g) | 0.99 [0.92, 1.07] | 0.779 | 0.764 | 0.98 [0.90, 1.07] | 0.793 | 0.755 | 57.1 |
| Total fat (g) | 0.96 [0.90, 1.03] | 0.849 | 0.873 | 1.00 [0.92, 1.09] | 0.715 | 0.716 | 68.3 |
| Saturated fat (g) | 0.96 [0.86, 1.07] | 0.826 | 0.813 | 1.04 [0.93, 1.17] | 0.728 | 0.732 | 68.3 |
| Polyunsaturated fat (g) | 0.77 [0.51, 1.14] | 0.754 | 0.638 | 0.95 [0.66, 1.37] | 0.724 | 0.572 | 73 |
| Retinol Vitamin A (mcg) | 1.23 [0.84, 1.79] | 0.841 | 0.743 | 0.96 [0.62, 1.49] | 0.836 | 0.534 | 73 |
| beta-Carotene (mcg) | 1.12 [0.90, 1.40] | 0.907 | 0.862 | 0.93 [0.68, 1.26] | 0.739 | 0.729 | 77.8 |
| Vitamin D (mcg) | 0.73 [0.49, 1.08] | 0.778 | 0.715 | 0.99 [0.72, 1.37] | 0.769 | 0.748 | 73 |
| Vitamin E (mg) | 0.97 [0.82, 1.13] | 0.682 | 0.789 | 0.87 [0.74, 1.02] | 0.709 | 0.665 | 60.3 |
| Vitamin C (mg) | 0.93 [0.78, 1.12] | 0.895 | 0.812 | 1.06 [0.91, 1.22] | 0.91 | 0.908 | 81 |
| Vitamin B1 (mg) | 0.84 [0.66, 1.07] | 0.67 | 0.676 | 0.92 [0.75, 1.12] | 0.774 | 0.742 | 63.5 |
| Vitamin B2 (mg) | 0.97 [0.89, 1.06] | 0.825 | 0.797 | 0.99 [0.90, 1.10] | 0.726 | 0.886 | 66.7 |
| Niacin (mg) | 1.00 [0.88, 1.13] | 0.77 | 0.762 | 0.97 [0.84, 1.12] | 0.736 | 0.626 | 66.7 |
| Pantothenic acid (mg) | 0.90 [0.80, 1.00] | 0.849 | 0.839 | 0.93 [0.82, 1.05] | 0.653 | 0.654 | 68.3 |
| Vitamin B6 (mg) | 0.99 [0.87, 1.13] | 0.82 | 0.794 | 0.90 [0.79, 1.04] | 0.735 | 0.684 | 68.3 |
| Biotin (mcg) | 0.93 [0.82, 1.05] | 0.791 | 0.872 | 0.96 [0.87, 1.06] | 0.821 | 0.859 | 63.5 |
| Folate (mcg) | 0.98 [0.87, 1.10] | 0.84 | 0.831 | 0.98 [0.86, 1.11] | 0.753 | 0.746 | 65.1 |
| Vitamin B12 (mcg) | 0.92 [0.60, 1.43] | 0.735 | 0.543 | 1.34 [0.94, 1.91] | 0.532 | 0.502 | 55.6 |
| Calcium (mg) | 0.98 [0.88, 1.10] | 0.761 | 0.775 | 1.07 [0.93, 1.23] | 0.667 | 0.666 | 76.2 |
| Magnesium (mg) | 1.02 [0.94, 1.10] | 0.742 | 0.718 | 0.98 [0.90, 1.08] | 0.75 | 0.662 | 58.7 |
| Iron (mg) | 0.97 [0.89, 1.05] | 0.743 | 0.764 | 1.00 [0.91, 1.09] | 0.714 | 0.703 | 63.5 |
| Phosphorus (mg) | 0.98 [0.91, 1.06] | 0.792 | 0.776 | 0.99 [0.90, 1.09] | 0.667 | 0.659 | 71.4 |
| Copper (mcg) | 1.04 [0.67, 1.62] | 0.714 | 0.582 | 0.90 [0.76, 1.07] | 0.801 | 0.89 | 74.6 |
| Zinc (mcg) | 0.95 [0.60, 1.49] | 0.793 | 0.673 | 0.98 [0.72, 1.33] | 0.819 | 0.839 | 71.4 |
| Potassium (mg) | 0.99 [0.93, 1.06] | 0.819 | 0.813 | 0.99 [0.91, 1.07] | 0.753 | 0.752 | 58.7 |
| Sodium (mg) | 0.95 [0.84, 1.08] | 0.828 | 0.758 | 0.99 [0.91, 1.08] | 0.776 | 0.817 | 74.6 |

Table S2. Association between the estimates of nutrient intake using the self-administered online 24-hour dietary recall and the interviewer-administered 24-hour dietary recall split by recall number and % observation in the same tertile (average of the two days). Cohort: Older school-age children (N=64).

| Nutrient | Recall 1 | | | Recall 2 | | | Observations in the same tertile |
| --- | --- | --- | --- | --- | --- | --- | --- |
|  | Geometric mean ratio [95% CI] | Spearman correlation coefficient (rho) | Intra-class correlation coefficient | Geometric mean ratio [95% CI] | Spearman correlation coefficient (rho) | Intra-class correlation coefficient | % |
| Energy (Kcal) | 0.95 [0.88, 1.02] | 0.789 | 0.761 | 0.92 [0.84, 1.01] | 0.86 | 0.769 | 76.6 |
| Protein (g) | 0.89 [0.81, 0.97] | 0.747 | 0.749 | 0.90 [0.79, 1.03] | 0.86 | 0.618 | 71.9 |
| Total sugar (g) | 0.90 [0.78, 1.03] | 0.742 | 0.693 | 0.93 [0.85, 1.02] | 0.807 | 0.836 | 64.1 |
| Carbohydrates (g) | 0.97 [0.90, 1.05] | 0.821 | 0.839 | 0.91 [0.84, 0.99] | 0.879 | 0.837 | 78.1 |
| Dietary fibers (g) | 0.94 [0.85, 1.04] | 0.807 | 0.832 | 0.91 [0.83, 1.00] | 0.853 | 0.793 | 62.5 |
| Total fat (g) | 0.92 [0.82, 1.02] | 0.737 | 0.698 | 0.88 [0.75, 1.03] | 0.851 | 0.669 | 75 |
| Saturated fat (g) | 0.91 [0.74, 1.11] | 0.802 | 0.54 | 0.90 [0.78, 1.04] | 0.777 | 0.759 | 76.6 |
| Polyunsaturated fat (g) | 0.97 [0.75, 1.25] | 0.797 | 0.792 | 0.85 [0.69, 1.05] | 0.862 | 0.886 | 75 |
| Retinol Vitamin A (mcg) | 0.32 [0.15, 0.68] | 0.709 | 0.637 | 0.98 [0.58, 1.65] | 0.943 | 0.78 | 76.6 |
| beta-Carotene (mcg) | 0.90 [0.50, 1.62] | 0.722 | 0.603 | 0.86 [0.56, 1.34] | 0.942 | 0.734 | 70.3 |
| Vitamin D (mcg) | 0.70 [0.44, 1.11] | 0.661 | 0.606 | 0.86 [0.55, 1.34] | 0.766 | 0.667 | 75 |
| Vitamin E (mg) | 1.00 [0.81, 1.23] | 0.818 | 0.744 | 0.98 [0.79, 1.22] | 0.814 | 0.733 | 73.4 |
| Vitamin C (mg) | 1.01 [0.81, 1.27] | 0.819 | 0.822 | 1.10 [0.88, 1.39] | 0.868 | 0.753 | 75 |
| Vitamin B1 (mg) | 1.02 [0.89, 1.18] | 0.74 | 0.779 | 0.93 [0.83, 1.04] | 0.914 | 0.831 | 71.9 |
| Vitamin B2 (mg) | 0.87 [0.75, 1.01] | 0.706 | 0.567 | 0.89 [0.80, 1.00] | 0.877 | 0.732 | 62.5 |
| Niacin (mg) | 0.93 [0.81, 1.08] | 0.756 | 0.746 | 0.86 [0.75, 0.99] | 0.685 | 0.77 | 67.2 |
| Pantothenic acid (mg) | 1.04 [0.84, 1.30] | 0.684 | 0.62 | 0.93 [0.79, 1.10] | 0.735 | 0.746 | 64.1 |
| Vitamin B6 (mg) | 1.04 [0.81, 1.33] | 0.69 | 0.704 | 0.92 [0.77, 1.09] | 0.811 | 0.803 | 70.3 |
| Biotin (mcg) | 0.94 [0.81, 1.09] | 0.77 | 0.76 | 1.00 [0.86, 1.16] | 0.777 | 0.774 | 68.8 |
| Folate (mcg) | 0.95 [0.82, 1.09] | 0.79 | 0.792 | 0.94 [0.78, 1.13] | 0.694 | 0.648 | 71.9 |
| Vitamin B12 (mcg) | 0.97 [0.72, 1.31] | 0.805 | 0.805 | 0.82 [0.61, 1.10] | 0.792 | 0.779 | 78.1 |
| Calcium (mg) | 0.92 [0.81, 1.04] | 0.896 | 0.87 | 0.89 [0.76, 1.05] | 0.827 | 0.659 | 71.9 |
| Magnesium (mg) | 0.97 [0.90, 1.05] | 0.858 | 0.83 | 0.98 [0.91, 1.07] | 0.85 | 0.752 | 67.2 |
| Iron (mg) | 0.87 [0.77, 0.98] | 0.708 | 0.634 | 0.89 [0.78, 1.02] | 0.86 | 0.683 | 67.2 |
| Phosphorus (mg) | 0.93 [0.85, 1.03] | 0.841 | 0.777 | 0.90 [0.80, 1.02] | 0.787 | 0.605 | 81.3 |
| Copper (mcg) | 1.09 [0.63, 1.86] | 0.747 | 0.646 | 0.72 [0.48, 1.07] | 0.734 | 0.637 | 73.4 |
| Zinc (mcg) | 0.72 [0.40, 1.31] | 0.788 | 0.717 | 0.69 [0.43, 1.09] | 0.801 | 0.701 | 76.6 |
| Potassium (mg) | 1.00 [0.90, 1.11] | 0.808 | 0.713 | 0.96 [0.89, 1.04] | 0.845 | 0.78 | 73.4 |
| Sodium (mg) | 0.97 [0.79, 1.21] | 0.773 | 0.584 | 0.74 [0.59, 0.93] | 0.782 | 0.478 | 70.3 |

Table S3. Association between the estimates of nutrient intake using the self-administered online 24-hour dietary recall and the interviewer-administered 24-hour dietary recall split by recall number and % observation in the same tertile (average of the two days). Cohort: Adults (N=67).

| Nutrient | Recall 1 | | | Recall 2 | | | Observations in the same tertile |
| --- | --- | --- | --- | --- | --- | --- | --- |
|  | Geometric mean ratio [95% CI] | Spearman correlation coefficient (rho) | Intra-class correlation coefficient | Geometric mean ratio [95% CI] | Spearman correlation coefficient (rho) | Intra-class correlation coefficient | % |
| Energy (Kcal) | 0.92 [0.87, 0.98] | 0.829 | 0.826 | 0.97 [0.92, 1.03] | 0.813 | 0.825 | 76.1 |
| Protein (g) | 0.93 [0.86, 1.01] | 0.871 | 0.833 | 0.97 [0.89, 1.04] | 0.748 | 0.788 | 74.6 |
| Total sugar (g) | 0.87 [0.77, 0.99] | 0.729 | 0.759 | 0.99 [0.89, 1.11] | 0.767 | 0.729 | 65.7 |
| Carbohydrates (g) | 0.92 [0.85, 0.98] | 0.842 | 0.799 | 0.96 [0.89, 1.02] | 0.826 | 0.822 | 65.7 |
| Dietary fibers (g) | 0.87 [0.77, 0.99] | 0.867 | 0.807 | 0.92 [0.84, 1.01] | 0.749 | 0.768 | 80.6 |
| Total fat (g) | 0.92 [0.86, 0.98] | 0.849 | 0.877 | 0.96 [0.90, 1.03] | 0.846 | 0.87 | 71.6 |
| Saturated fat (g) | 0.88 [0.77, 1.00] | 0.855 | 0.785 | 0.96 [0.87, 1.06] | 0.857 | 0.874 | 76.1 |
| Polyunsaturated fat (g) | 0.85 [0.57, 1.26] | 0.662 | 0.661 | 0.95 [0.74, 1.20] | 0.85 | 0.907 | 62.7 |
| Retinol Vitamin A (mcg) | 0.88 [0.69, 1.12] | 0.944 | 0.909 | 0.74 [0.52, 1.07] | 0.794 | 0.748 | 83.6 |
| beta-Carotene (mcg) | 0.99 [0.72, 1.38] | 0.802 | 0.766 | 0.63 [0.38, 1.04] | 0.748 | 0.601 | 73.1 |
| Vitamin D (mcg) | 0.87 [0.63, 1.19] | 0.832 | 0.795 | 0.92 [0.68, 1.24] | 0.876 | 0.816 | 74.6 |
| Vitamin E (mg) | 0.89 [0.76, 1.05] | 0.774 | 0.778 | 0.98 [0.83, 1.15] | 0.742 | 0.762 | 67.2 |
| Vitamin C (mg) | 0.72 [0.54, 0.97] | 0.816 | 0.667 | 0.90 [0.76, 1.07] | 0.85 | 0.889 | 83.6 |
| Vitamin B1 (mg) | 0.76 [0.59, 0.99] | 0.614 | 0.37 | 0.88 [0.67, 1.16] | 0.71 | 0.3 | 65.7 |
| Vitamin B2 (mg) | 0.83 [0.69, 1.01] | 0.794 | 0.411 | 1.00 [0.90, 1.10] | 0.76 | 0.733 | 67.2 |
| Niacin (mg) | 0.92 [0.79, 1.07] | 0.842 | 0.739 | 0.93 [0.85, 1.02] | 0.793 | 0.838 | 71.6 |
| Pantothenic acid (mg) | 0.76 [0.58, 1.01] | 0.758 | 0.629 | 0.84 [0.66, 1.07] | 0.721 | 0.492 | 74.6 |
| Vitamin B6 (mg) | 0.74 [0.55, 1.00] | 0.811 | 0.594 | 0.86 [0.64, 1.15] | 0.707 | 0.392 | 73.1 |
| Biotin (mcg) | 0.95 [0.83, 1.08] | 0.851 | 0.847 | 0.96 [0.84, 1.11] | 0.812 | 0.826 | 82.1 |
| Folate (mcg) | 0.87 [0.75, 1.00] | 0.827 | 0.772 | 0.93 [0.81, 1.07] | 0.761 | 0.721 | 71.6 |
| Vitamin B12 (mcg) | 0.95 [0.67, 1.36] | 0.809 | 0.736 | 0.98 [0.77, 1.25] | 0.868 | 0.869 | 73.1 |
| Calcium (mg) | 0.92 [0.81, 1.04] | 0.767 | 0.787 | 0.97 [0.89, 1.06] | 0.802 | 0.846 | 67.2 |
| Magnesium (mg) | 0.90 [0.83, 0.99] | 0.881 | 0.737 | 0.97 [0.89, 1.06] | 0.707 | 0.69 | 76.1 |
| Iron (mg) | 0.92 [0.85, 0.99] | 0.859 | 0.856 | 0.96 [0.88, 1.04] | 0.781 | 0.791 | 70.1 |
| Phosphorus (mg) | 0.90 [0.83, 0.98] | 0.813 | 0.767 | 0.96 [0.90, 1.03] | 0.811 | 0.839 | 74.6 |
| Copper (mcg) | 0.64 [0.42, 0.97] | 0.746 | 0.658 | 0.78 [0.55, 1.09] | 0.67 | 0.777 | 79.1 |
| Zinc (mcg) | 0.57 [0.34, 0.96] | 0.829 | 0.767 | 0.74 [0.48, 1.14] | 0.794 | 0.804 | 77.6 |
| Potassium (mg) | 0.91 [0.82, 1.01] | 0.851 | 0.716 | 0.96 [0.89, 1.03] | 0.814 | 0.802 | 76.1 |
| Sodium (mg) | 0.94 [0.85, 1.04] | 0.801 | 0.818 | 0.94 [0.87, 1.02] | 0.827 | 0.883 | 74.6 |

Table S4. Daily energy and nutrient intakes recorded by participants using the self-administered online 24-hour dietary recall and the interviewer-administered 24-hour dietary recall split by recall number. Cohort: Younger school age children (N=63).

| Nutrient | Recall 1 |  |  | Recall 2 |  |  |
| --- | --- | --- | --- | --- | --- | --- |
|  | Online self-administered | Interviewer-administered |  | Online self-administered | Interviewer-administered |  |
|  | Mean (SD) | Mean (SD) | *P*-value ^a^ | Mean (SD) | Mean (SD) | *P*-value ^a^ |
| Energy (Kcal) | 1695.1 (587.4) | 1712 (568.2) | 0.88^b^ | 1680.4 (528.8) | 1660.7 (552.1) | .63 |
| Protein (g) | 64.9 (24.7) | 65.2 (21.5) | 0.7^b^ | 63.5 (20.9) | 65.1 (23.1) | .67 |
| Total sugar (g) | 76.8 (39.9) | 81.7 (39.6) | 0.16 | 75.7 (41.8) | 72.2 (34.1) | .95 |
| Carbohydrates (g) | 204.2 (69.9) | 206.6 (72.7) | 0.85^b^ | 197.1 (73.2) | 191.7 (68.3) | .58 |
| Dietary fibers (g) | 12.7 (5.7) | 12.8 (5.7) | 0.83 | 12.5 (6.2) | 12.8 (7.2) | .69 |
| Total fat (g) | 70.6 (35.4) | 73.2 (35.4) | 0.52^b^ | 73.5 (33.9) | 74.4 (34.8) | .99 |
| Saturated fat (g) | 21.3 (13.3) | 22.6 (14.1) | 0.44^b^ | 24.7 (13.3) | 24.2 (11.8) | .48 |
| Polyunsaturated fat (g) | 4.3 (4.2) | 4.8 (4.3) | 0.19 | 4.4 (3.4) | 5 (5) | .78 |
| Retinol Vitamin A (mcg) | 267.8 (436.2) | 354.2 (832.8) | 0.28 | 298.6 (410.5) | 305.3 (376.8) | .86 |
| beta-Carotene (mcg) | 931.6 (1223.1) | 860.5 (1213.6) | 0.3 | 1095.6 (1796.1) | 1214.8 (2158.6) | .63 |
| Vitamin D (mcg) | 1.5 (2.9) | 1.7 (3.5) | 0.11 | 1.4 (1.6) | 1.5 (1.7) | .96 |
| Vitamin E (mg) | 7 (7.8) | 7.3 (7.3) | 0.66 | 6.1 (5.5) | 6.4 (4.8) | .09 |
| Vitamin C (mg) | 63.2 (62.1) | 65.4 (63.8) | 0.43 | 53.8 (51.2) | 51.9 (52.6) | .46 |
| Vitamin B1 (mg) | 4.6 (23.4) | 6.2 (23.6) | 0.15 | 2.3 (6.9) | 5.3 (17.6) | .40 |
| Vitamin B2 (mg) | 1.1 (0.6) | 1.1 (0.6) | 0.5 | 2 (3.2) | 2.4 (5.8) | .87 |
| Niacin (mg) | 12.3 (11.3) | 12.5 (10.5) | 0.95 | 10.7 (6.7) | 11.2 (6.8) | .64 |
| Pantothenic acid (mg) | 3.3 (3.6) | 3.5 (3.2) | 0.06 | 3.3 (2.8) | 3.4 (2.6) | .24 |
| Vitamin B6 (mg) | 1.1 (1.2) | 1.1 (1) | 0.88 | 1 (0.7) | 1 (0.7) | .16 |
| Biotin (mcg) | 35 (80.5) | 36.2 (71.8) | 0.23 | 28.8 (48.8) | 29 (44.3) | .38 |
| Folate (mcg) | 109.5 (114.4) | 111.1 (104.3) | 0.73 | 104.9 (85.1) | 105.1 (86.5) | .75 |
| Vitamin B12 (mcg) | 7.1 (14.1) | 4.7 (9.7) | 0.71 | 9.3 (15.4) | 5.5 (10.5) | .11 |
| Calcium (mg) | 683.1 (436) | 660.5 (377.4) | 0.75 | 724 (407.7) | 654.8 (345.5) | .33 |
| Magnesium (mg) | 202 (85.7) | 197.8 (84) | 0.69 | 202.5 (76.9) | 204.8 (82.2) | .72 |
| Iron (mg) | 8.5 (4.1) | 8.7 (4.2) | 0.27^b^ | 8.7 (3.4) | 9 (3.9) | .93 |
| Phosphorus (mg) | 939.4 (397.5) | 935.9 (377.4) | 0.66 | 976.1 (388.5) | 979.2 (367) | .86 |
| Copper (mcg) | 355.3 (428.5) | 320.9 (360.2) | 0.86 | 274.5 (220.3) | 325.5 (294.4) | .22 |
| Zinc (mcg) | 2208.2 (2392.2) | 1947.9 (2315.1) | 0.81 | 2082.1 (1953.7) | 2213.3 (1998.7) | .88 |
| Potassium (mg) | 1984.6 (761.9) | 1978 (727.5) | 0.85 | 1931.4 (822.1) | 1924.7 (791.6) | .79 |
| Sodium (mg) | 2641.5 (1574.7) | 2809.2 (1813.8) | 0.42 | 2532.2 (1258.3) | 2582.3 (1327.3) | .85 |

^a^Paired samples t-test (Shapiro Wilk P<0.05)

^b^Wilcoxon signed rank test used (Shapiro Wilk P>0.05)

Table S5. Daily energy and nutrient intakes recorded by participants using the online self-administered 24-hour dietary recall and the interviewer-administered 24-hour dietary recall split by recall number. Cohort: Older school age children (N=64).

| Nutrient | Recall 1 |  |  | Recall 2 |  |  |
| --- | --- | --- | --- | --- | --- | --- |
|  | Online self-administered | Interviewer-administered |  | Online self-administered | Interviewer-administered |  |
|  | Mean (SD) | Mean (SD) | *P*-value ^a^ | Mean (SD) | Mean (SD) | *P*-value ^a^ |
| Energy (Kcal) | 2025.3 (1049.6) | 2077.9 (913.3) | 0.17 | 1757.1 (765.9) | 1820.6 (737.9) | .07 |
| Protein (g) | 78.8 (42.7) | 86.5 (45.2) | 0.01 | 73.4 (36.8) | 74.5 (30.5) | .14 |
| Total sugar (g) | 75.9 (59.8) | 74.5 (40.5) | 0.13 | 60.9 (35.9) | 65.4 (41.3) | .12 |
| Carbohydrates (g) | 220.9 (134.4) | 221.7 (115.4) | 0.42 | 180 (89.1) | 192.6 (97.8) | .02 |
| Dietary fibers (g) | 12.8 (7.4) | 14.1 (9.4) | 0.31^b^ | 11.4 (7.3) | 11.6 (6) | .05 |
| Total fat (g) | 93.2 (56.6) | 96.8 (47) | 0.11 | 82.7 (44.5) | 84.5 (41.8) | .11 |
| Saturated fat (g) | 27.1 (21.1) | 27.8 (18.5) | 0.34 | 23.7 (14.6) | 23.4 (12.2) | .14 |
| Polyunsaturated fat (g) | 6.9 (6.4) | 7 (6.8) | 0.82 | 5 (5.3) | 4.8 (4.6) | .12 |
| Retinol Vitamin A (mcg) | 278.4 (521.4) | 667.4 (2600.8) | 0 | 336.6 (507.9) | 380.1 (670.9) | .93 |
| beta-Carotene (mcg) | 577.2 (847.1) | 633 (917.6) | 0.73 | 693.9 (1213.4) | 732.3 (1264.4) | .50 |
| Vitamin D (mcg) | 1.2 (1.5) | 1.2 (1.5) | 0.13 | 1.5 (1.8) | 1.5 (1.9) | .50 |
| Vitamin E (mg) | 5.9 (5.6) | 6.4 (6.1) | 0.98 | 5.3 (4.5) | 5.3 (4.9) | .85 |
| Vitamin C (mg) | 43.9 (48.2) | 44.3 (46.4) | 0.91 | 45 (40.9) | 45 (42.3) | .39 |
| Vitamin B1 (mg) | 2.5 (12.9) | 1.5 (4.3) | 0.75 | 0.9 (0.8) | 0.9 (0.8) | .22 |
| Vitamin B2 (mg) | 1.3 (0.8) | 1.5 (0.8) | 0.06 | 1.2 (0.7) | 1.3 (0.7) | .06 |
| Niacin (mg) | 15.2 (10.6) | 17.3 (12.9) | 0.23^b^ | 13.2 (10.2) | 14.1 (9.7) | .04 |
| Pantothenic acid (mg) | 3.5 (2.5) | 3.4 (2.4) | 0.7 | 3.2 (3.3) | 3.1 (2.6) | .40 |
| Vitamin B6 (mg) | 1.1 (0.8) | 1.1 (0.9) | 0.75 | 1 (0.9) | 1 (1) | .32 |
| Biotin (mcg) | 24.5 (30.4) | 24.5 (28.2) | 0.4 | 28.7 (43) | 26.3 (38.3) | .95 |
| Folate (mcg) | 96.1 (87.6) | 102.7 (90.4) | 0.43 | 90.1 (78.1) | 98.7 (95.5) | .49 |
| Vitamin B12 (mcg) | 6.7 (14.8) | 6.2 (13) | 0.85 | 4.5 (8.9) | 4.7 (9.8) | .18 |
| Calcium (mg) | 686.5 (626.7) | 689.3 (495.5) | 0.11^b^ | 608.6 (366.2) | 623 (326.2) | .16 |
| Magnesium (mg) | 209.3 (110.3) | 216.5 (112.4) | 0.4^b^ | 188.5 (83.1) | 186.6 (71.4) | .68 |
| Iron (mg) | 9.4 (5.3) | 10.9 (6.4) | 0.02 | 8.3 (4.6) | 9.1 (5) | .09 |
| Phosphorus (mg) | 1067.8 (595) | 1130.9 (570.4) | 0.17 | 965.2 (460.1) | 1002.4 (389.7) | .09 |
| Copper (mcg) | 273.5 (324.6) | 282.5 (340.9) | 0.76 | 236.7 (255.9) | 248.4 (272.2) | .10 |
| Zinc (mcg) | 1915.7 (2795) | 2319.4 (3413.1) | 0.28 | 1481.1 (1704) | 1493.9 (1921.4) | .11 |
| Potassium (mg) | 2191.9 (1064.7) | 2231.7 (1034.3) | 0.99 | 1982.1 (988.1) | 2004.5 (846.2) | .33 |
| Sodium (mg) | 2567.5 (1864.8) | 2750.1 (2091.7) | 0.8 | 2171.3 (1534.1) | 2442.1 (1157.8) | .01 |

^a^Paired samples t-test (Shapiro Wilk P<0.05)

^b^Wilcoxon signed rank test used (Shapiro Wilk P>0.05)

Table S6. Daily energy and nutrient intakes recorded by participants using the self-administered online 24-hour dietary recall and the interviewer-administered 24-hour dietary recall split by recall number. Cohort: Adults (N=67).

| Nutrient | Recall 1 |  |  | Recall 2 |  |  |
| --- | --- | --- | --- | --- | --- | --- |
|  | Online self-administered | Interviewer-administered |  | Online self-administered | Interviewer-administered |  |
|  | Mean (SD) | Mean (SD) | *P*-value ^a^ | Mean (SD) | Mean (SD) | *P*-value ^a^ |
| Energy (Kcal) | 1870.9 (956.9) | 1995.1 (1010.6) | .02 | 1867.4 (785.4) | 1891 (723.8) | 0.33 |
| Protein (g) | 83 (46.1) | 86.3 (44.5) | .09 | 77.8 (35.3) | 79 (32.9) | 0.37 |
| Total sugar (g) | 55.2 (35) | 60.9 (39.2) | .04 | 56.9 (42.4) | 53.9 (28.8) | 0.89 |
| Carbohydrates (g) | 174.2 (84.3) | 185.8 (86.5) | .02 | 186.6 (90.2) | 192.3 (88.3) | 0.18 |
| Dietary fibers (g) | 13.4 (7.8) | 14.2 (7.6) | .03 | 12.9 (6.5) | 13.9 (6.7) | 0.08 |
| Total fat (g) | 92 (65) | 98.7 (68.1) | .02 | 92.6 (53.1) | 92.5 (49.4) | 0.28 |
| Saturated fat (g) | 23.6 (19.7) | 25.8 (22) | .05 | 26.2 (23.1) | 25.7 (21.8) | 0.38 |
| Polyunsaturated fat (g) | 5.3 (6) | 6 (6.3) | .41 | 4.9 (5.5) | 5.4 (6.3) | 0.65 |
| Retinol Vitamin A (mcg) | 318.3 (457.4) | 287.8 (350.9) | .29 | 290 (344.3) | 299 (332.5) | 0.11 |
| beta-Carotene (mcg) | 1232.7 (3427.2) | 1082.8 (2369.1) | .96 | 1262.9 (2204.1) | 1266 (1937.1) | 0.07 |
| Vitamin D (mcg) | 2.1 (3.6) | 2.1 (3.8) | .38 | 2.6 (4.6) | 2.6 (4.9) | 0.58 |
| Vitamin E (mg) | 7.8 (9.1) | 7.9 (7.6) | .17 | 7 (5.3) | 7 (5.3) | 0.79 |
| Vitamin C (mg) | 56.4 (65.3) | 59.1 (59.5) | .03 | 49.8 (61.4) | 50.8 (68.2) | 0.24 |
| Vitamin B1 (mg) | 0.9 (1) | 5.7 (27) | .04 | 3.2 (18.8) | 3.2 (13.1) | 0.37 |
| Vitamin B2 (mg) | 1.4 (0.8) | 2.8 (8.6) | .06 | 1.3 (0.7) | 1.3 (0.7) | 0.95 |
| Niacin (mg) | 19 (18.4) | 18.6 (14.7) | .28 | 15.6 (10.2) | 16.8 (11.1) | 0.13 |
| Pantothenic acid (mg) | 18.5 (84.1) | 27.5 (102.8) | .06 | 3 (2.5) | 10.9 (62.7) | 0.16 |
| Vitamin B6 (mg) | 5 (20.3) | 8.1 (28.3) | .05 | 1.1 (0.9) | 3 (15.1) | 0.32 |
| Biotin (mcg) | 43.1 (94.5) | 36.9 (57.5) | .43 | 30.9 (43.2) | 31.9 (47.6) | 0.6 |
| Folate (mcg) | 148 (167.9) | 162.5 (175.1) | .06 | 119.2 (110.7) | 121.2 (110) | 0.33 |
| Vitamin B12 (mcg) | 7.6 (16.3) | 6.2 (12.5) | .79 | 5.3 (11.3) | 4.7 (11.4) | 0.88 |
| Calcium (mg) | 628.1 (400.4) | 650.4 (383.8) | .20 | 607.9 (361.7) | 606 (311.5) | 0.6^b^ |
| Magnesium (mg) | 226.5 (107.1) | 239.9 (103.1) | .03 | 214.1 (91.6) | 216.8 (86.6) | 0.49 |
| Iron (mg) | 10.2 (6.1) | 11 (6.2) | .03 | 10 (4.6) | 10.3 (4.4) | 0.27 |
| Phosphorus (mg) | 1074.1 (528.7) | 1151.8 (516.7) | .02 | 1021.8 (458.7) | 1042.2 (404.8) | 0.21^b^ |
| Copper (mcg) | 333.4 (349.3) | 398.1 (393.8) | .04 | 320.4 (314.5) | 339.9 (328.8) | 0.15 |
| Zinc (mcg) | 2095.7 (2522) | 2458.4 (2857) | .04 | 1761.5 (2022.6) | 1856.4 (2168.1) | 0.17 |
| Potassium (mg) | 2297.4 (1218.5) | 2424.3 (1144.5) | .06 | 2043.3 (980) | 2073.8 (862.5) | 0.24 |
| Sodium (mg) | 2420 (1465.8) | 2528.9 (1472.4) | .21 | 2872.5 (1580.5) | 3037.7 (1680.3) | 0.14^b^ |

^a^Paired samples t-test (Shapiro Wilk P<0.05)

^b^Wilcoxon signed rank test used (Shapiro Wilk P>0.05)
